# Addition of Capecitabine to Adjuvant Chemotherapy May be the Most Effective Strategy for Patients With Early-Stage Triple-Negative Breast Cancer: A Network Meta-Analysis of 9 Randomized Controlled Trials

**DOI:** 10.3389/fendo.2022.939048

**Published:** 2022-07-11

**Authors:** Zhiyang Li, Jiehua Zheng, Zeqi Ji, Lingzhi Chen, Jinyao Wu, Juan Zou, Yiyuan Liu, Weixun Lin, Jiehui Cai, Yaokun Chen, Yexi Chen, Hai Lu

**Affiliations:** ^1^ Department of Breast, The First People's Hospital of Shao Guan, Shaoguan, China; ^2^ Department of Thyroid, Breast and Hernia Surgery, The Second Affiliated Hospital of Shantou University Medical College, Shantou, China

**Keywords:** triple-negative breast cancer, capecitabine, adjuvant chemotherapy, neoadjuvant chemotherapy, network meta-analysis

## Abstract

**Background and Objective:**

Previous studies determined the therapeutic effects of capecitabine-based chemotherapy regimens on early-stage triple-negative breast cancer (TNBC). However, the optimal strategy of capecitabine-based chemotherapy remains uncertain. We conducted this network meta-analysis to address this issue.

**Methods:**

We systematically searched PubMed, Embase, and the Cochrane Registry of Controlled Trials (CENTRAL) to retrieve eligible studies published before September 2021. Two independent reviewers extracted information from eligible studies using a pre-designed data extraction sheet. The primary outcome included disease-free survival, and the second outcome showed overall survival and adverse events. Direct meta-analysis was performed using RevMan 5.4, and Bayesian network analysis was performed using R version 3.6.1 with the “gemtc” and “rjags” packages.

**Results:**

Nine studies involving 3661 TNBC patients met the selection criteria. The network meta-analysis suggested that the addition of capecitabine to adjuvant chemotherapy achieved a significantly longer disease-free (HR = 0.66, 95% CrI = 0.49 to 0.86) and overall survival time (HR = 0.60, 95% CrI = 0.43 to 0.83) than standard chemotherapy. All comparisons did not achieve statistical significance. The addition of capecitabine to adjuvant chemotherapy was the most effective treatment for improving disease-free (81.24%) and overall survival (78.46%) times, and the replacement of capecitabine to adjuvant chemotherapy was the safest regime.

**Conclusions:**

Based on available evidence, capecitabine-based chemotherapy benefits TNBC patients, and the addition of capecitabine with adjuvant chemotherapy was the most effective regime. In contrast, the replacement of capecitabine to adjuvant chemotherapy was the safest regime. More studies of high quality and large scale are needed to confirm our findings.

## 1 Introduction

Breast cancer (BC) has the highest incidence among cancers worldwide (1). It is pointed out that, among all subtypes of breast cancers (2), triple-negative breast cancer (TNBC) accounts for approximately 12 to 17% of all breast cancers (3). TNBC refers to the absence of amplification of estrogen receptor (ER)—negative, progesterone receptor (PR)—negative, and human epidermal growth factor receptor 2 (HER2)—negative (4), which was characterized by a higher recurrence rate and shorter overall survival (OS) (5). Although standard chemotherapy containing anthracycline and taxane was recommended for the preventing recurrence and survival improvement among early TNBC patients (6–8), a proportion of TNBC patients eventually undergo from recurrence regardless of tumor stage (9). It is, therefore, crucial to investigate novel adjuvant strategies for improving the prognosis of TNBC patients.

As an oral pro-drug of fluorouracil, capecitabine has been approved for treating metastatic BC patients who experienced progression after anthracyclines and taxanes (10). Several randomized clinical trials (RCTs) investigating the effectiveness and safety of capecitabine-based chemotherapeutic regimes in treating TNBC (11–16) reported conflicting results. Moreover, four published meta-analyses (17–20) solely or separately considered early TNBC patients; however, they reported inconsistent results. Moreover, the optimal strategy for early TNBC patients remains unclear because a direct comparison of different capecitabine-based chemotherapeutic regimes is missing.

Although pair-wise meta-analysis provides a method for comprehensively investigating the comparative effectiveness and safety of two interventions, it is impossible to determine the relative differences between two interventions that were not directly compared. Meanwhile, pair-wise meta-analysis cannot simultaneously determine the comparative effectiveness of multiple interventions (more than 2) at one time. Fortunately, as an expansion of pairwise meta-analysis, network meta-analysis can simultaneously compare multiple interventions at one time and achieve the probabilities of the relative rank of all interventions (21). Therefore, we conducted this study to investigate the comparative efficacy and safety of different capecitabine-based chemotherapeutic regimes for TNBC using the network meta-analysis technique.

## 2 Materials and Methods

This network meta-analysis was in line with the preferred reporting items for systematic reviews and meta-analysis (PRISMA) for network meta-analysis (PRISMA-NMA) (22, 23) and the Cochrane Collaboration recommendations (24).

### 2.1 Search Strategy

Two reviewers independently searched the following electronic databases for eligible studies for relevant studies published before September 2021, including PubMed, Embase, and the Cochrane Registry of Controlled Trials (CENTRAL). Meanwhile, we also checked the reference lists of topic-related reviews and included studies to identify any eligible studies. The complete search strategy is summarized in **Table S1**. The latest search was updated just before the final analysis. Any disagreements were resolved by agreeing with a third reviewer.

### 2.2 Eligibility Criteria

The inclusion criteria were: (a) patients with early-stage TNBC were involved and separately analyzed, (b) TNBC patients in one arm received standard chemotherapy and patients in the other arm received capecitabine-based chemotherapeutic regimes, including replacement or addition of capecitabine to adjuvant chemotherapy and replacement of capecitabine to neoadjuvant chemotherapy, (c) studies reported hazard ratios (HRs) with 95% confidence interval (CI) of disease-free survival (DFS) (or RFS) and overall survival (OS) or had sufficient data for calculating HRs with 95% CIs, and odds ratio (OR) with 95% CI of any grade 3–4 adverse events (AEs) or had sufficient data for calculating OR with 95% CI, and (d) randomized controlled trial was published in English and full-text. The chemotherapeutic regime was a neoadjuvant strategy when concomitant use of adjuvant and neoadjuvant chemotherapy was applied in our network meta-analysis. Studies were excluded if there were (a) non-RCT, (b) studies focusing on metastatic breast cancer, or (c) studies with insufficient data.

### 2.3 Study Selection

First, we used EndNote software to remove duplicate studies. Second, two independent reviewers initially screened the titles and abstracts of all retained studies to check their eligibility. Third, the full texts of potentially eligible studies were retrieved and reviewed by two independent reviewers. Any disagreements were resolved by agreeing with a third reviewer.

### 2.4 Data Extraction

Two independent reviewers extracted essential data from the eligible studies using standard data extraction tables. The main information of each eligible study, namely, study design, population characteristics and sample size, details of therapeutic regimes, outcomes, and information on methodological quality, was extracted. Necessary information for the final analysis was added by contacting the lead author. Any disagreements were resolved by agreeing with a third reviewer.

### 2.5 Definition of Outcomes

In this network meta-analysis, DFS was the primary outcome, and OS and adverse events were the secondary outcomes. DFS was defined as the time from randomization to the date of diagnosis of locoregional or distant recurrence, the second primary malignancy, or death. We used recurrence-free survival (RFS), which is defined as the time from randomization to the date of recurrence or death, to replace DFS when the value for DFS was not provided. OS is defined as the time from randomization to death due to any cause. Adverse events were assessed according to the National Cancer Institute Common Terminology Criteria for Adverse Events Version 3.0.

### 2.6 Quality Assessment

The risk of bias of each eligible study was independently assessed by two reviewers using the Cochrane risk of bias assessment tool (25). Six domains were assessed to determine individual studies as having a low, unclear, or high risk, including selection bias, performance bias, detection bias, attrition bias, reporting bias, and other bias sources. The risk bias summary of each study was conducted using Review Manager 5.4 (Review Manager, the Cochrane Collaboration, 2020).

### 2.7 Statistical Analysis

#### 2.7.1 Methods for Direct Meta-Analysis

A conventional direct meta-analysis was performed using RevMan software to calculate the relative direct effects of the competitive regimes. A random-effects model was used to calculate all estimates because variations were available for all studies in the real settings (26, 27). We used HR with 95% CI to express the estimates of DFS and OS, and OR with 95% CI to express the estimates of adverse events. For all statistical analyses, P <0.05 implied a significant effect size.

#### 2.7.2 Methods for Network Meta-Analysis

We performed a Bayesian network analysis based on the Markov chain Monte Carlo (MCMC) (28) to investigate the comparative effectiveness and safety of different capecitabine-based chemotherapy regimens. The burn-in period was defined as 20,000 simulations for each chain, and posterior summaries are based on 50,000 subsequent simulations (29). We visually assessed the convergence of chains by inspecting traces and density plots (30). Moreover, we also used the potential scale reduced factor (PSRF), which was obtained from Brooks–Gelman–Rubin plots, to assess the convergence of the chains (31, 32). The results from the network meta-analysis were expressed as HR or OR with a 95% credible interval (CrI) (33), which was summarized as a league figure (34). We calculated the relative rank probability of all regimes in terms of each outcome to determine the optimal option (35). Statistical analysis was performed using R software (V.3.6.1) (36) and the packages “gemtc” and “rjags” (37).

#### 2.7.3 Methods of Assessing Heterogeneity, Inconsistency, and Small Study Effects

We used I2 statistics to assess statistical heterogeneity in the conventional pairwise meta-analysis (38) and used the heterogeneity variance parameter (τ2) (39) to assess the global heterogeneity of the network meta-analysis models by using the mtc.anohe command. Unfortunately, it was impossible to locally investigate inconsistencies between direct and indirect effects by using the split-node method because direct evidence was not available for all comparisons (40, 41). Moreover, we did not also estimate publication bias due to the insufficient number of eligible studies (26).

## 3 Results

### 3.1 Study Selection

**Figure 1:**
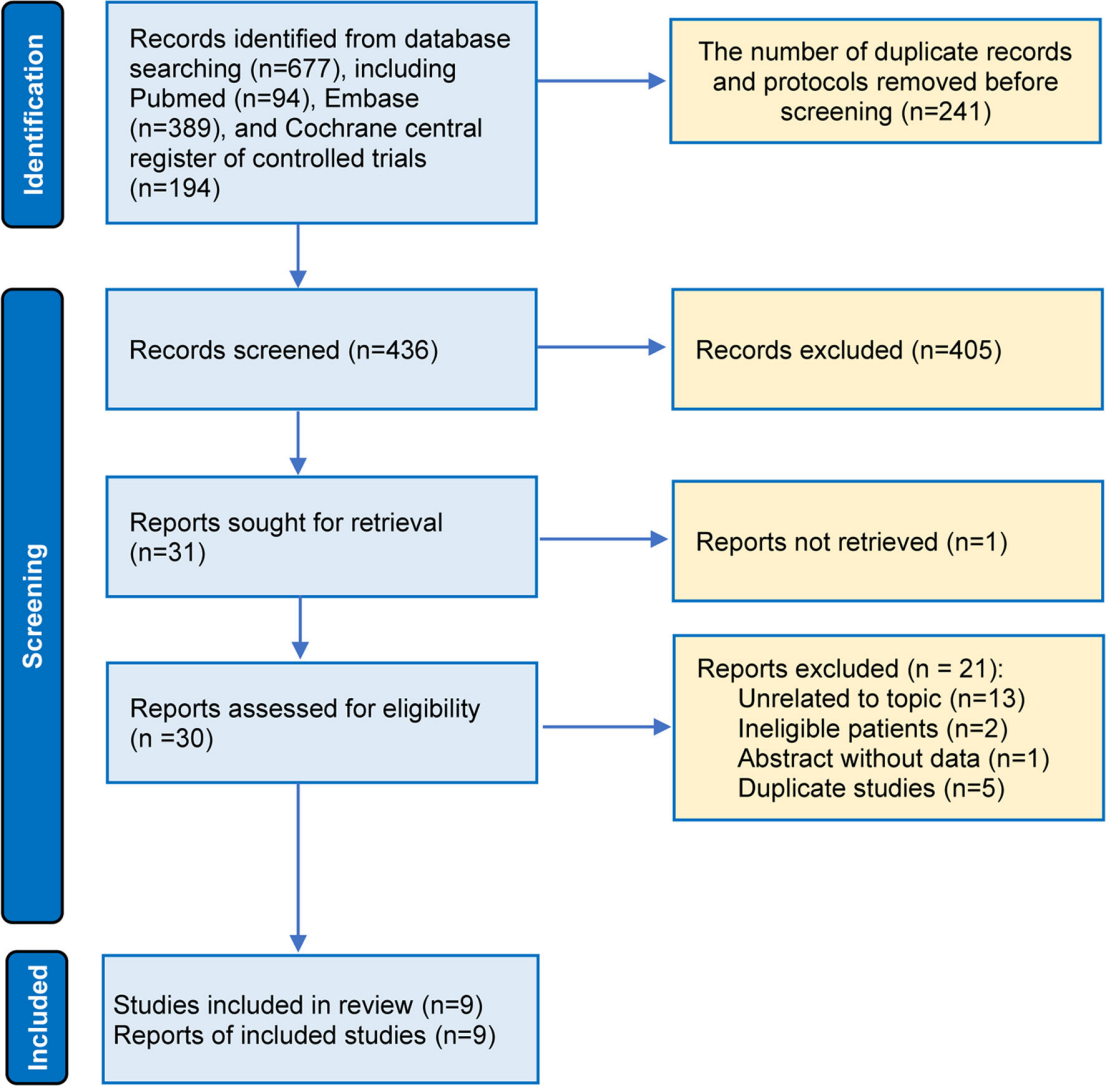
Flow diagram of the study retrieval and selection.

Among 111 identified studies, 26 records were removed as duplicate studies. Then, we excluded 58 ineligible studies after screening the titles and abstracts. The full-texts of 26 articles were downloaded, and 17 studies were excluded for several reasons, as follows: unrelated to our topic (n = 9), ineligible patients (n = 2), abstracts without sufficient data (n = 1), and duplicate studies (n = 5) (**Figure 1**). Finally, 9 studies (11–16) were included in the final analysis, reporting information on 3 comparisons among 3 different capecitabine-based chemotherapy regimes (addition of capecitabine to adjuvant chemotherapy [AA], replacement of capecitabine to adjuvant chemotherapy [RA], and replacement of capecitabine to neoadjuvant chemotherapy [RNA]) (**Figure S1**). It is necessary to say that we updated the latest results of the Fin XX study (42) in our data analysis because it has been reported during the peer review period.

### 3.2 Characteristics of Included Studies

Among the 9 included studies, 2 studies (15, 16) enrolled TNBC patients as the entire cohort, while TNBC patients were analyzed as one subgroup in the remaining 7 studies (11–14, 43–45). All studies (11–16) were updated between 2015 and 2020, with an accumulated sample size of 3661. Eight studies (11, 13–16, 43–45) reported HR values for disease-free survival except for 1 study (12), which reported the HR values for recurrence-free survival. The main characteristics of 9 eligible studies are shown in **Table 1**. Moreover, we designed **Table 2** to summarize the reported odds ratios of adverse events.

**Table 1:** Basic characteristics of all eligible studies.

Study	Update year	Trial phase	Capecitabine arm	Control arm	Population	TNBC, N(X vs Control)	Median Follow-up, years	Design	Reported HR	
									DFS	OS
FinXX	2017/2022	III	TX-CEX	T-CEF	Subgroup	93 vs 109	10.3	AA	0.53 (0.31, 0.92)	0.59 (0.36, 0.97)
GEICAM/2003-10	2015	III	ET-X	EC-T	Subgroup	95 vs 71	6.6	RA	1.19 (0.70, 2.04)	n.a.
USO-01062	2015	III	AC-TX	AC-T	Subgroup	396 vs 384	5.0	AA	0.81 (0.57, 1.15)	0.62 (0.41, 0.94)
CREATE-X	2017	III	X	None	Subgroup	139 vs 147	5.0	AA	0.58 (0.39, 0.87)	0.52 (0.30, 0.90)
CIBOMA-2004/01	2020	III	ED-X	EC-T	Whole cohort	353 vs 352	7.3	RNA	0.77 (0.59, 1.00)	0.86 (0.63, 1.20)
Gepar TRIO	2013	III	TAC-NX	TAC-TAC	Subgroup	362	5.2	RNA	0.87 (0.61, 1.25)	n.a.
GAIN	2017	III	EC-PX	EPC	Subgroup	213 vs 208	5.0	RA	0.97 (0.68, 1.38)	0.81 (0.54, 1.20)
CBCSG-010	2020	III	TX-XEC	T-FEC	Whole cohort	297 vs 288	5.6	AA	0.66 (0.44, 0.99)	0.67 (0.37, 1.22)
CALGB49907	2019	III	X	CMF-AC	Subgroup	76 vs 78	11.4	RA	0.67 (0.44, 1.00)	0.71 (0.45, 1.14)

X, capecitabine; C, cyclophosphamide; M, methotrexate; F, 5-fluorouracil; A, anthracycline; E, epirubicin; T, docetaxel; P, paclitaxel; N, nab-paclitaxe; AA, addition of capecitabine to adjuvant chemotherapy; RA, replacement of capecitabine to adjuvant chemotherapy; RNA, replacement of capecitabine to neoadjuvant chemotherapy; SCT, standard chemotherapy; HR, hazard ratio; DFS, disease-free survival; OS, overall survival; n.a., not applicable.

**Table 2:** Reported odds ratios of adverse events in included studies.

Study	Capecitabine arm	Control arm	Design	OR for AEs	
				Any	Grade 3–4
USO-01062	AC-TX	AC-T	AA	8.88 (0.48, 165.02)	1.53 (1.17, 2.00)
CIBOMA-2004/01	ED-X	EC-T	RNA	11.82 (7.24, 19.30)	3.72 (2.69, 5.14)
GAIN	EC-PX	EPC	RA	1.20 (0.51, 2.77)	0.46 (0.38, 0.55)
CBCSG-010	TX-XEC	T-FEC	AA	0.77 (0.26, 2.24)	0.90 (0.59, 1.38)

X, capecitabine; C, cyclophosphamide; F, 5-fluorouracil; A, anthracycline; E, epirubicin; T, docetaxel; P, paclitaxel; AA, addition of capecitabine to adjuvant chemotherapy; RA, replacement of capecitabine to adjuvant chemotherapy; RNA, replacement of capecitabine to neoadjuvant chemotherapy; OR, odds ratio.

### 3.3 Quality Assessment

**Figure 2:**
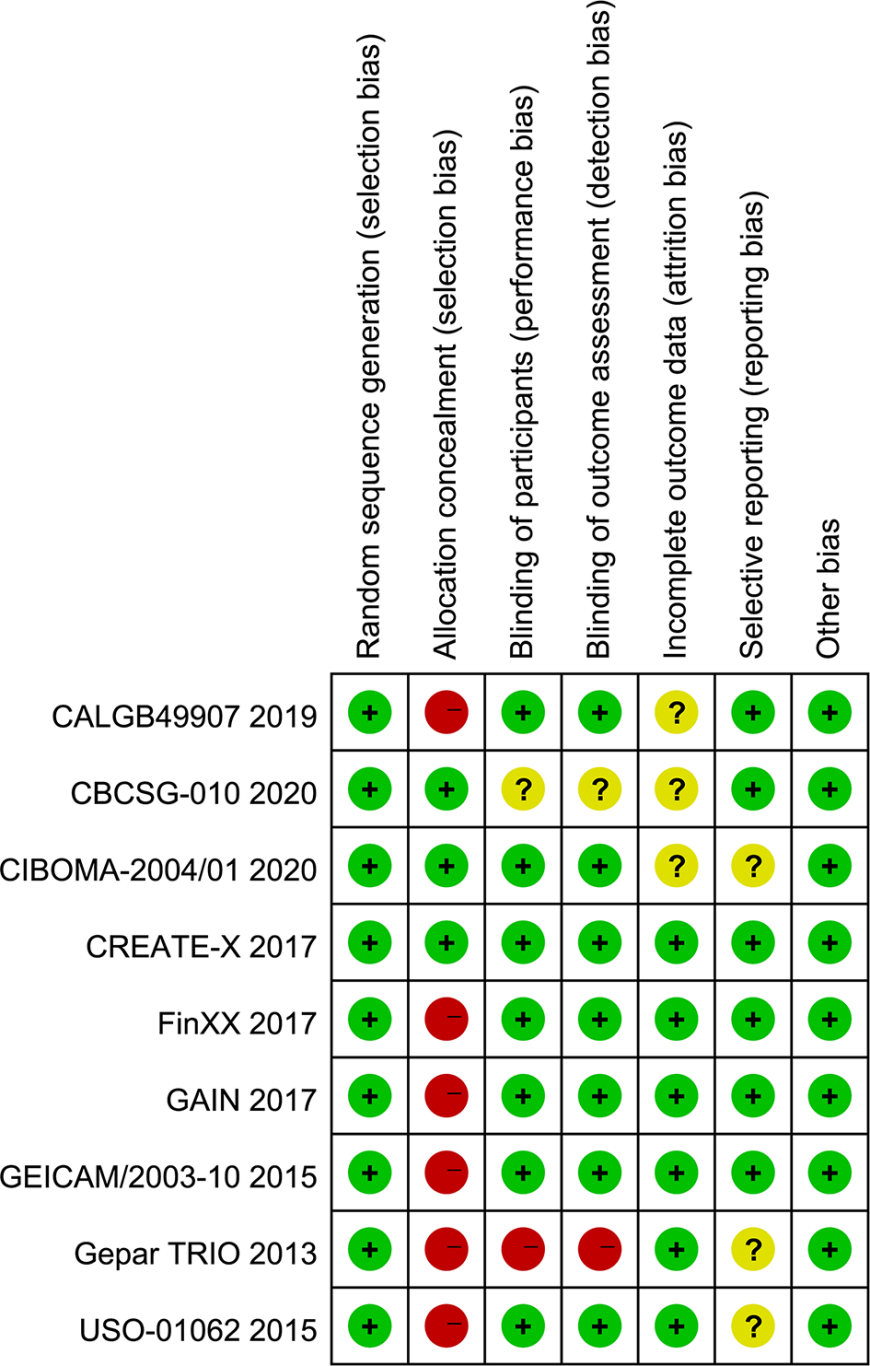
Authors’ judgments of each risk of bias item for eligible studies.

The results of the quality assessment of each study are displayed in **Figure 2**. Overall, the quality levels of most included studies (11, 12, 14–16, 43–45) were appraised as low to moderate, except for 1 study (13), which was appraised as high. Among 8 eligible studies with a low quality level, 75% (11, 12, 14, 43–45) did not correctly conduct allocation concealment and 12.5% (43) did not appropriately blind personnel and outcome assessor.

### 3.4 Disease-Free Survival

All included studies reported HR values for disease-free survival or recurrence-free survival. Direct meta-analysis indicated that capecitabine-based chemotherapy had a clearly longer disease-free survival time than standard chemotherapy (HR = 0.77, 95% CI = 0.67 to 0.88, I^2^ = 15%). Subgroup analysis further revealed that benefit was provided only by the addition of capecitabine to adjuvant chemotherapy (HR = 0.66, 95% CI = 0.54 to 0.82, I^2^ = 0%) and replacement of capecitabine with neoadjuvant chemotherapy (HR = 0.80, 95% CI = 0.65 to 0.99, I^2^ = 0%). However, network meta-analysis only showed that the addition of capecitabine to adjuvant chemotherapy significantly prolonged disease-free survival time (HR = 0.66, 95% CrI = 0.49 to 0.86). The results of direct and network meta-analysis are illustrated in **Figures S2**, **3A**, respectively. Rank probability indicated that the addition of capecitabine to adjuvant chemotherapy ranked first (81.24%), followed by the replacement of capecitabine to neoadjuvant chemotherapy (54.13%) and the replacement of capecitabine to adjuvant chemotherapy (46.12%) (**Figure 4A**).

**Figure 3:**
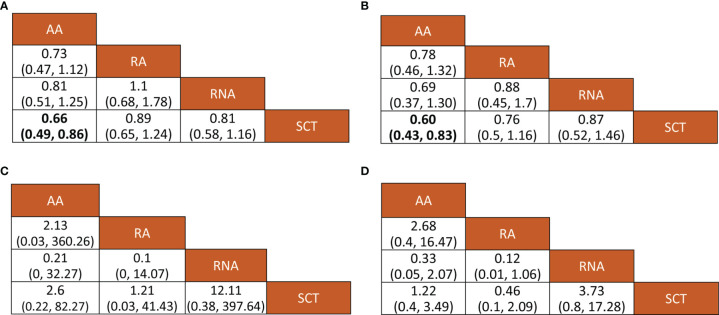
Relative effects of various outcomes. Bold numerical values indicate significant pairwise comparison. **(A)** Disease-free survival, **(B)** overall survival; **(C)** any adverse events, and **(D)** grade 3–4 adverse events. AA, addition of capecitabine to adjuvant chemotherapy; RA, replacement of capecitabine to adjuvant chemotherapy; RNA, replacement of capecitabine to neoadjuvant chemotherapy; SCT, standard chemotherapy.

**Figure 4:**
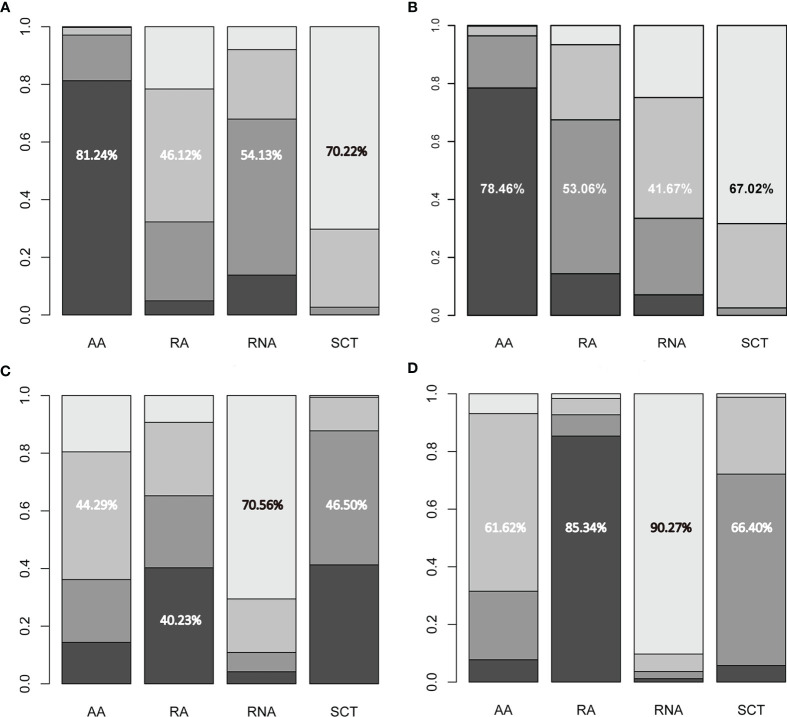
Ranking probabilities of available regimes for **(A)** disease-free survival, **(B)** overall survival, **(C)** any adverse events, and **(D)** grade 3–4 adverse events. The numerical values indicate the probability of ranking at certain places. AA, addition of capecitabine to adjuvant chemotherapy; RA, replacement of capecitabine to adjuvant chemotherapy; RNA, replacement of capecitabine to neoadjuvant chemotherapy; SCT, standard chemotherapy.

### 3.5 Overall Survival

Seven studies involving 3,133 patients reported HR values for overall survival. Direct meta-analysis suggested that capecitabine-based chemotherapy significantly prolonged overall survival time than that of standard chemotherapy (HR = 0.71, 95% CI = 0.60 to 0.84, I^2^ = 0%), however subgroup analysis revealed that benefit was provided only by the addition of capecitabine to adjuvant chemotherapy (HR = 0.60, 95% CI = 0.47 to 0.77, I^2^ = 0%), which was also supported by network meta-analysis (HR = 0.60, 95% CrI = 0.43 to 0.83). The results of the direct and network meta-analysis are illustrated in **Figures S3**, **3B**, respectively. Rank probability indicated that the addition of capecitabine to adjuvant chemotherapy ranked first (78.46%), followed by the replacement of capecitabine to adjuvant chemotherapy (53.06%), and the replacement of capecitabine to neoadjuvant chemotherapy (41.67%) (**Figure 4B**).

### 3.6 Any Adverse Events

Among 9 eligible studies, 4 studies reported the incidence of any adverse events. Direct meta-analysis suggested no statistical difference between capecitabine-based chemotherapy and standard chemotherapy (OR = 2.85, 95% CI = 0.54 to 15.08, I^2^ = 91%). However, subgroup analysis revealed that more adverse events occurred in patients treated by replacement of capecitabine with neoadjuvant chemotherapy (OR = 11.82, 95% CI = 7.24 to 19.30, I^2^ = NA) (**Figure S4A**). Nevertheless, network meta-analysis did not detect differences among all comparisons (**Figure 3C**). Rank probability indicated that replacement of capecitabine to neoadjuvant chemotherapy was worst (70.56%), followed by the addition of capecitabine to adjuvant chemotherapy (44.29%) and replacement of capecitabine to adjuvant chemotherapy (40.23%) (**Figure 4C**).

### 3.7 Grade 3–4 Adverse Events

A total 4 studies reported the incidence of grade 3–4 adverse events. Direct meta-analysis suggested no statistical difference between capecitabine-based chemotherapy and standard chemotherapy (OR = 1.23, 95% CI = 0.47 to 3.21, I^2^ = 98%). However, subgroup analysis revealed that more adverse events occurred in patients treated by replacement of capecitabine with neoadjuvant chemotherapy (OR = 3.72, 95% CI = 2.69 to 5.14, I^2^ = NA). In contrast, patients treated by replacement of capecitabine with adjuvant chemotherapy reported fewer grade 3–4 adverse events than patients receiving standard chemotherapy (OR = 0.46, 95% CI = 0.38 to 0.55, I^2^ = NA) (**Figure S4B**). Nevertheless, network meta-analysis did not detect differences among all comparisons (**Figure 3D**). Rank probability indicated that replacement of capecitabine to neoadjuvant chemotherapy was worst (90.27%), followed by the addition of capecitabine to adjuvant chemotherapy (61.62%) and replacement of capecitabine to adjuvant chemotherapy (85.34%) (**Figure 4D**).

### 3.8 Results of Inconsistency, Publication Bias, and Heterogeneity Analyses

The inconsistency assessment did not apply to our network meta-analysis due to the absence of a closed loop in the network plot. Meanwhile, publication bias (small sample effects) was also not applicable to our network meta-analysis owing to an insufficient accumulated number of eligible studies. The I^2^ value for the entire network, which was generated from the heterogeneity analysis, was 2.0% (DFS), 0% (OS), 21.0% (any adverse events), and 15.0% (grade 3–4 adverse events), indicating no significant statistical heterogeneity.

### 3.9 Convergence Assessment

**Figure 5:**
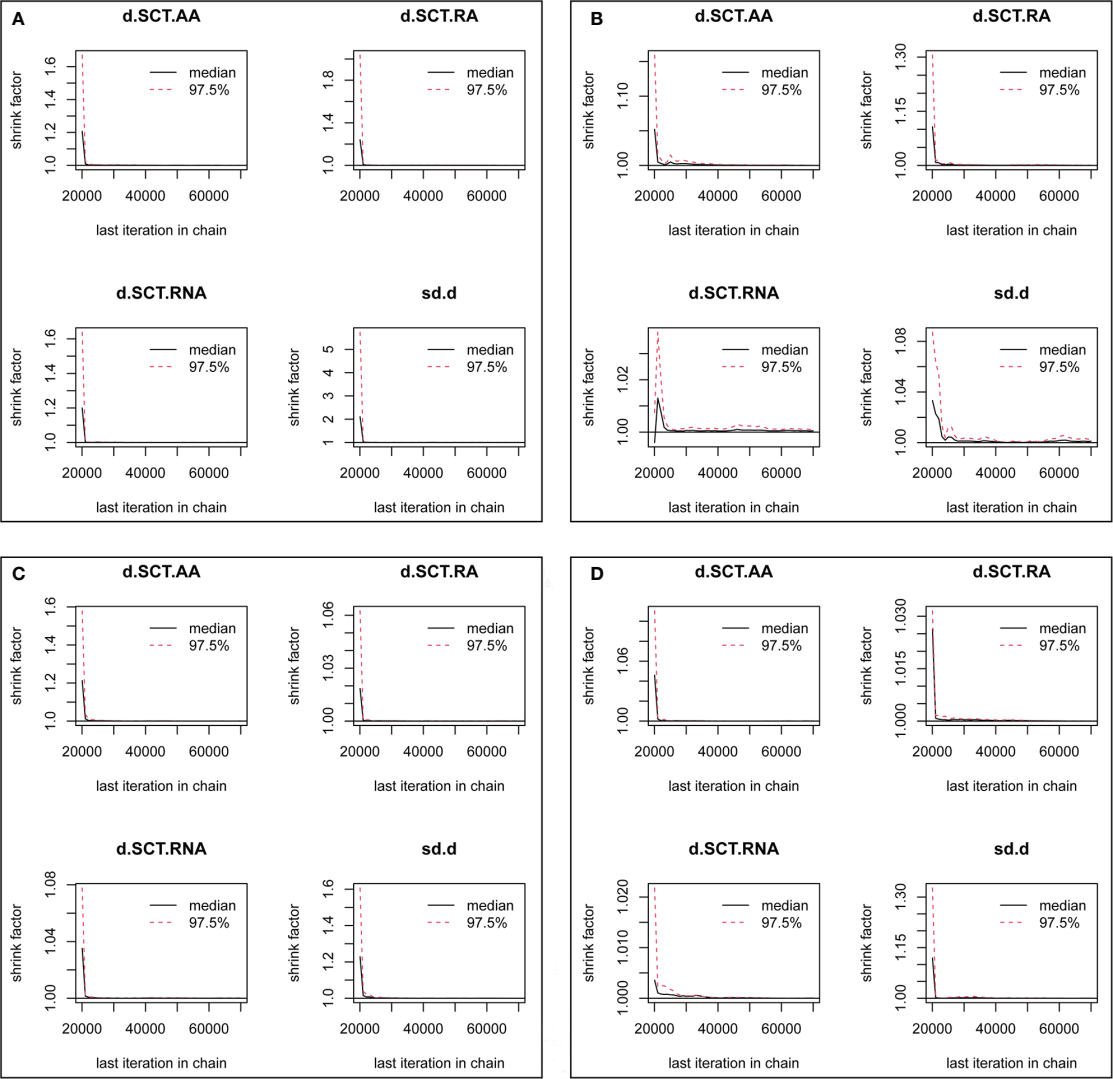
Convergence assessment based on gelman plot for each outcome. **(A)** Disease-free survival, **(B)** overall survival, **(C)** any adverse events, and **(D)** grade 3–4 adverse events. AA, addition of capecitabine to adjuvant chemotherapy; RA, replacement of capecitabine to adjuvant chemotherapy; RNA, replacement of capecitabine to neoadjuvant chemotherapy; SCT, standard chemotherapy.

After running 50,000 iterations, the trace plot suggested that stable fusion was achieved for each MCMC chain, and a single chain could not be distinguished by the naked eye. Meanwhile, a density plot suggesting a smooth normal distribution curve was generated for each direct comparison, and the bandwidth was close to zero and stable, with values ranging from 0.01 to 0.05. The full results of the trace and density plots can be found in **Figure S5**. Moreover, the Brooks–Gelman–Rubin diagnostic plot suggested that the shrink factor for each analysis was close to 1.0 and stable, as shown in **Figure 5**. Overall, the results of trace, density, and diagnostic plots suggested a good convergence.

## 4 Discussion

The treatment of TNBC has been particularly emphasized because it accounts for approximately 12–17% of all breast cancers (3) and has higher recurrence rates and shorter OS (5). To improve the prognosis of early TNBC patients, capecitabine-based chemotherapeutic regimes have been clinically investigated (11–16, 43–45). Meanwhile, several meta-analyses have also been performed to focus on this issue and determine the clinical value of capecitabine-based chemotherapeutic regimes. Unfortunately, a conclusive finding has not yet been generated about which capecitabine-based chemotherapeutic regime may be the most effective and safe option.

To the best of our knowledge, this is the first network meta-analysis to investigate the comparative effectiveness and safety of different capecitabine-based chemotherapeutic regimes based on the most comprehensive studies. In this network meta-analysis, we first confirmed that capecitabine-based chemotherapeutic regimes had a clearly longer disease-free survival time and overall survival time compared with standard chemotherapy, which was consistent with the results of previous meta-analyses (17–19). Meanwhile, among 9 eligible studies, 2 studies (15, 16) which solely enrolled TNBC patients also consistently suggested a longer disease-free survival time in patients receiving capecitabine-based chemotherapeutic regimes compared with standard chemotherapy. Moreover, these 2 studies (15, 16) also revealed a numerically longer overall survival time in patients receiving capecitabine-based chemotherapeutic regimens, although there was no statistical difference. Certainly, insufficient sample size (1,290) may be the main contributor to the conflicting results with our network meta-analysis, in which a sample size of 3,133 was accumulated. Furthermore, our study simultaneously summarized direct and indirect evidence to enhance the robustness of our results.

Moreover, we also evaluated the safety of different capecitabine-based chemotherapeutic regimes in TNBC patients. The results from our network meta-analysis did not detect a statistical difference when comparing one regime with another, regardless of overall or grade 3–4 adverse events. In our network meta-analysis, we calculated the risk of experiencing adverse events among overall patients rather than a single TNBC cohort, which was also used in a previous meta-analysis (19). Although a previous meta-analysis separately reported the risk of adverse events according to systems, it generated similar results to our network meta-analysis, suggesting that capecitabine-based chemotherapeutic regimes resulted in tolerable adverse events. Another previous meta-analysis (18) only reported the incidence of adverse events in TNBC patients, which should be cautiously interpreted because the data were reported in limited studies. Among 4 included studies (14–16, 44), conflicting results were also reported. The USO-01062 (44) and CIBOMA-2004/01 (16) trials indicated that the addition of capecitabine to adjuvant chemotherapy significantly increased the risk of grade 3–4 adverse events, and the addition of capecitabine to neoadjuvant chemotherapy significantly increased the risk of overall adverse events and grade 3–4 adverse events. However, the CBCSG-010 trial (15) did not detect an increase in risk of adverse events in patients receiving the addition of capecitabine to adjuvant chemotherapy. Obviously, 8 cycles of capecitabine were applied in the USO-01062 (44) and CIBOMA-2004/01 (16) trials, which were significantly longer than 3 cycles of capecitabine in the CBCSG-010 trial (15). Moreover, the GAIN trial (14) suggested a decreased risk of grade 3–4 adverse events, which may be mainly because the replacement strategy reduced accumulated chemotherapy-related toxicity compared with the addition strategy (19). Additionally, a recent meta-analysis included 13 studies to investigate the effects of capecitabine as part of neo-/adjuvant chemotherapy in breast cancer patients (20). This meta-analysis separately investigated the effects and safety of adding or replacing capecitabine to systematic CT and focused on TNBC patients by designing subgroups. It is necessary to note that some studies were included in this meta-analysis inappropriately because of patients confirmed as HER2+, such as ICE II (46). More importantly, this meta-analysis could not evaluate the relative effects and safety of these two strategies (i.e., addition or replacement of capecitabine). Certainly, this meta-analysis did not also differentiate neoadjuvant CT from adjuvant CT. Therefore, this network meta-analysis may generate more informative and convincing findings than the previous meta-analysis.

Compared to previous meta-analyses and clinical trials, our network meta-analysis also generated 2 additional promising findings. First, our network meta-analysis detected that the addition of capecitabine to an adjuvant chemotherapy strategy may be the most effective therapeutic regime, although three capecitabine-based chemotherapeutic regimes were comparable for disease-free survival and overall survival. Second, the replacement of capecitabine with an adjuvant chemotherapy strategy may be the safest therapeutic regime, although no statistical difference was detected between the 3 capecitabine-based chemotherapeutic regimes.

Several promising findings were generated from our network meta-analysis due to some methodological strength. First, we performed an updated search to identify potential studies based on previous meta-analyses, which contributed to reducing the risk of retrieval bias. Second, we determined the comparative effectiveness and safety of different capecitabine-based chemotherapeutic regimes based on indirect comparisons, although direct comparisons were not available. Third, we determined the most effective and safe capecitabine-based chemotherapeutic regime by calculating the rank probabilities of three capecitabine-based chemotherapeutic regimes. Forth, a good model convergence was achieved on the basis of the results of trace, density and diagnostic plots (gelman plot), which ensure the accuracy of all pooled results.

We must recognize that some limitations may impair the robustness of the network meta-analysis result. First, there was no direct comparison between different capecitabine-based chemotherapeutic regimes, which obviously limits the accuracy of pooled results. Second, the median follow-up time varied between 5.0 and 11.4 years, which may contribute to the false credibility of overall survival. Third, we evaluated the safety of different capecitabine-based chemotherapeutic regimes based on the adverse events of the entire cohort because the influence of adverse events related to the hormone receptor and HER-2 status was small. For this reason, the influence of other confounding factors, such as variations in chemotherapy regime and number of treatment cycles on, pooled results could not be investigated because of inadequate data.

## 5 Conclusion

Based on the results from our network meta-analysis, the addition of capecitabine-based chemotherapy can prolong both disease-free time and overall survival time as the most effective capecitabine-based chemotherapeutic regime. However, the replacement of capecitabine-based chemotherapy is the safest capecitabine-based chemotherapeutic regime. Unfortunately, no study was available for direct comparison between different capecitabine-based chemotherapeutic regimes. Therefore, further studies with rigor, methodological quality, and large-scale are needed to confirm their effectiveness.

## Data Availability Statement

The original contributions presented in the study are included in the article/**Supplementary Material**. Further inquiries can be directed to the corresponding authors.

## Author Contributions

Substantially contributed to conception or design: YXC and HL. Contributed to acquisition, analysis, or interpretation of data: ZL, JHZ, ZJ, and LC. Drafted the manuscript for important content: JZ, YL, and WL. Critically revised the manuscript for important intellectual content: JC, YKC, and JW. All authors listed have made a substantial, direct, and intellectual contribution to the work and approved it for publication.

## Funding

This work was supported by the Special Fund Project of Guangdong Science and Technology (210728156901524, 210728156901519), the Medical Scientific Research Foundation of Guangdong Province, China (grant number A2021432, B2021448), the Shantou Medical Science and Technology Planning Project (grant number 210521236491457, 210625106490696, 220518116490772, 220518116490933), and the Administration of Traditional Chinese Medicine of Guangdong Province project (202205092315428030).

## Conflict of Interest

The authors declare that the research was conducted in the absence of any commercial or financial relationships that could be construed as a potential conflict of interest.

## Publisher’s Note

All claims expressed in this article are solely those of the authors and do not necessarily represent those of their affiliated organizations, or those of the publisher, the editors and the reviewers. Any product that may be evaluated in this article, or claim that may be made by its manufacturer, is not guaranteed or endorsed by the publisher.
