# Supplementary material for: Addition of Capecitabine to Adjuvant Chemotherapy May be the Most Effective Strategy for Patients With Early-Stage Triple-Negative Breast Cancer: A Network Meta-Analysis of 9 Randomized Controlled Trials
Source: Front Endocrinol (Lausanne). 2022 Jul 11;13:939048. doi: 10.3389/fendo.2022.939048 (PMC9358934; doi:10.3389/fendo.2022.939048)
Supplement: Supplementary file 1 [file DataSheet_1.docx]

**Pubmed**

| **NO.** | **Query** | | **Results** |
| --- | --- | --- | --- |
| 10 | #9 and #6 and #7 | | 94 |
| 9 | #8 and #2 | | 43,599 |
| 8 | (early-stage[Title/Abstract]) OR (early[Title/Abstract]) | | 1,687,920 |
| 7 | random |  | 1,345,145 |
| 6 | #4 or #5 | | 8,084 |
| 5 | (Xeloda[Title/Abstract]) OR (Capecitabine[Title/Abstract]) | | 7,102 |
| 4 | "Capecitabine"[Mesh] | | 5,044 |
| 3 | #1 or #2 | | 419,703 |
| 2 | ((((((((((((((((((Breast Neoplasm[Title/Abstract]) OR (Breast Neoplasms[Title/Abstract])) OR (Breast Tumor[Title/Abstract])) OR (Breast Tumors[Title/Abstract])) OR (Breast Cancer[Title/Abstract])) OR (Breast Cancers[Title/Abstract])) OR (Mammary Cancer[Title/Abstract])) OR (Mammary Cancers[Title/Abstract])) OR (Breast Malignant Neoplasm[Title/Abstract])) OR (Breast Malignant Neoplasms[Title/Abstract])) OR (Malignant Tumor of Breast[Title/Abstract])) OR (Breast Malignant Tumor[Title/Abstract])) OR (Breast Malignant Tumors[Title/Abstract])) OR (Human Mammary Carcinoma[Title/Abstract])) OR (Human Mammary Carcinomas[Title/Abstract])) OR (Human Mammary Neoplasm[Title/Abstract])) OR (Human Mammary Neoplasms[Title/Abstract])) OR (Breast Carcinoma[Title/Abstract])) OR (Breast Carcinomas[Title/Abstract]) | | 341,803 |
| 1 | "Breast Neoplasms"[Mesh] | | 320,384 |

**Embase**

#11. #10 AND [embase]/lim 389

#10. #5 AND #8 AND #9 398

#9. random* 1,998,895

#8. #6 OR #7 34,262

#7. 'capecitabine'/exp 32,981

#6. xeloda:ti,ab,kw OR capecitabine:ti,ab,kw 14,362

#5. #3 AND #4 75,569

#4. 'early stage':ti,ab,kw OR early:ti,ab,kw 2,310,929

#3. #1 OR #2 670,549

#2. 'breast tumor'/exp OR 'triple negative breast 615,763

cancer'/exp

#1. 'breast neoplasm':ti,ab,kw OR 'breast 491,095

neoplasms':ti,ab,kw OR 'breast tumor':ti,ab,kw OR

'breast tumors':ti,ab,kw OR 'breast

cancer':ti,ab,kw OR 'breast cancers':ti,ab,kw OR

'mammary cancer':ti,ab,kw OR 'mammary

cancers':ti,ab,kw OR 'breast malignant

neoplasm':ti,ab,kw OR 'breast malignant

neoplasms':ti,ab,kw OR 'breast malignant

tumor':ti,ab,kw OR 'breast malignant

tumors':ti,ab,kw OR 'human mammary

carcinoma':ti,ab,kw OR 'human mammary

carcinomas':ti,ab,kw OR 'human mammary

neoplasm':ti,ab,kw OR 'human mammary

neoplasms':ti,ab,kw OR 'breast

carcinoma':ti,ab,kw OR 'breast

carcinomas':ti,ab,kw

**Cochrane Central Register of Controlled Trials**

ID Search Hits

#1 (Breast Neoplasm):ti,ab,kw OR (Breast Neoplasms):ti,ab,kw OR (Breast Tumor):ti,ab,kw OR (Breast Tumors):ti,ab,kw OR (Breast Cancer):ti,ab,kw 41073

#2 (Breast Cancers):ti,ab,kw OR (Mammary Cancer):ti,ab,kw OR (Mammary Cancers):ti,ab,kw OR (Breast Malignant Neoplasm):ti,ab,kw OR (Breast Malignant Neoplasms):ti,ab,kw 4389

#3 (Breast Malignant Tumor):ti,ab,kw OR (Breast Malignant Tumors):ti,ab,kw OR (Human Mammary Carcinoma):ti,ab,kw OR (Human Mammary Carcinomas):ti,ab,kw OR (Human Mammary Neoplasm):ti,ab,kw 995

#4 (Human Mammary Neoplasms):ti,ab,kw OR (Breast Carcinoma):ti,ab,kw OR (Breast Carcinomas):ti,ab,kw 4183

#5 #1 or #2 or #3 or #4 41496

#6 MeSH descriptor: [Breast Neoplasms] explode all trees 14205

#7 #5 or #6 41496

#8 (Xeloda):ti,ab,kw OR (Capecitabine):ti,ab,kw 4264

#9 MeSH descriptor: [Capecitabine] explode all trees 1350

#10 #8 or #9 4264

#11 (early-stage):ti,ab,kw OR (early):ti,ab,kw 129115

#12 #7 and #11 7435

#13 #12 and #10 194
