# Supplementary material for: Addition of Capecitabine to Adjuvant Chemotherapy May be the Most Effective Strategy for Patients With Early-Stage Triple-Negative Breast Cancer: A Network Meta-Analysis of 9 Randomized Controlled Trials
Source: Front Endocrinol (Lausanne). 2022 Jul 11;13:939048. doi: 10.3389/fendo.2022.939048 (PMC9358934; doi:10.3389/fendo.2022.939048)
Supplement: Supplementary file 2 [file DataSheet_2.docx]

**Figure legends**

**Figure S1.** Network plots of regimes in patients with early-stage TNBC for (a) disease-free survival, (b) overall survival, (c) any adverse events, and (d) grade 3-4 adverse events. The width of each line which indicates the direct comparison of two regimes is proportional to the number of included studies, and the size of each node is proportional to the number of accumulated sample size. AA, addition of capecitabine to adjuvant chemotherapy; RA, replacement of capecitabine to adjuvant chemotherapy; RNA, replacement of capecitabine to neoadjuvant chemotherapy; SCT, standard chemotherapy

**Figure S2.** Direct meta-analysis of the hazard ratios for disease-free survival based on different pairwise comparisons.

**Figure S3.** Direct meta-analysis of the hazard ratios for overall survival based on different pairwise comparisons.

**Figure S4.** Direct meta-analysis of the odds ratios for (a) any adverse events and (b) grade 3-4 adverse events based on different pairwise comparisons.

**Figure S5.** Trace and density plots of MCMC simulation for each regime. (a) disease-free survival, (b) overall survival, (c) any adverse events, and (d) grade 3-4 adverse events. AA, addition of capecitabine to adjuvant chemotherapy; RA, replacement of capecitabine to adjuvant chemotherapy; RNA, replacement of capecitabine to neoadjuvant chemotherapy; SCT, standard chemotherapy. MCMC, Markov chain Monte Carlo.


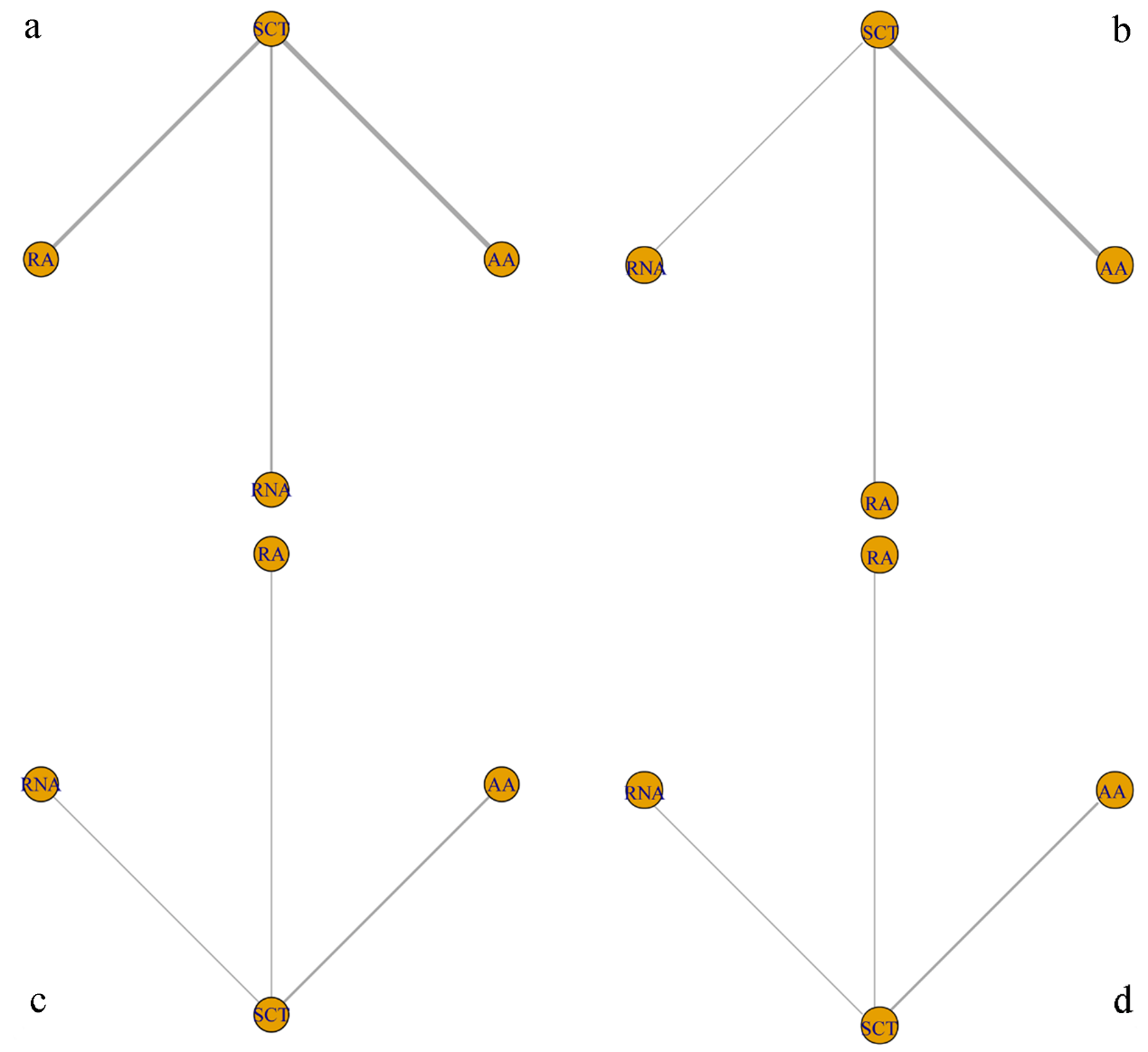


**Figure S1.** Network plots of regimes in patients with early-stage TNBC for (a) disease-free survival, (b) overall survival, (c) any adverse events, and (d) grade 3-4 adverse events. The width of each line which indicates the direct comparison of two regimes is proportional to the number of included studies, and the size of each node is proportional to the number of accumulated sample size. AA, addition of capecitabine to adjuvant chemotherapy; RA, replacement of capecitabine to adjuvant chemotherapy; RNA, replacement of capecitabine to neoadjuvant chemotherapy; SCT, standard chemotherapy


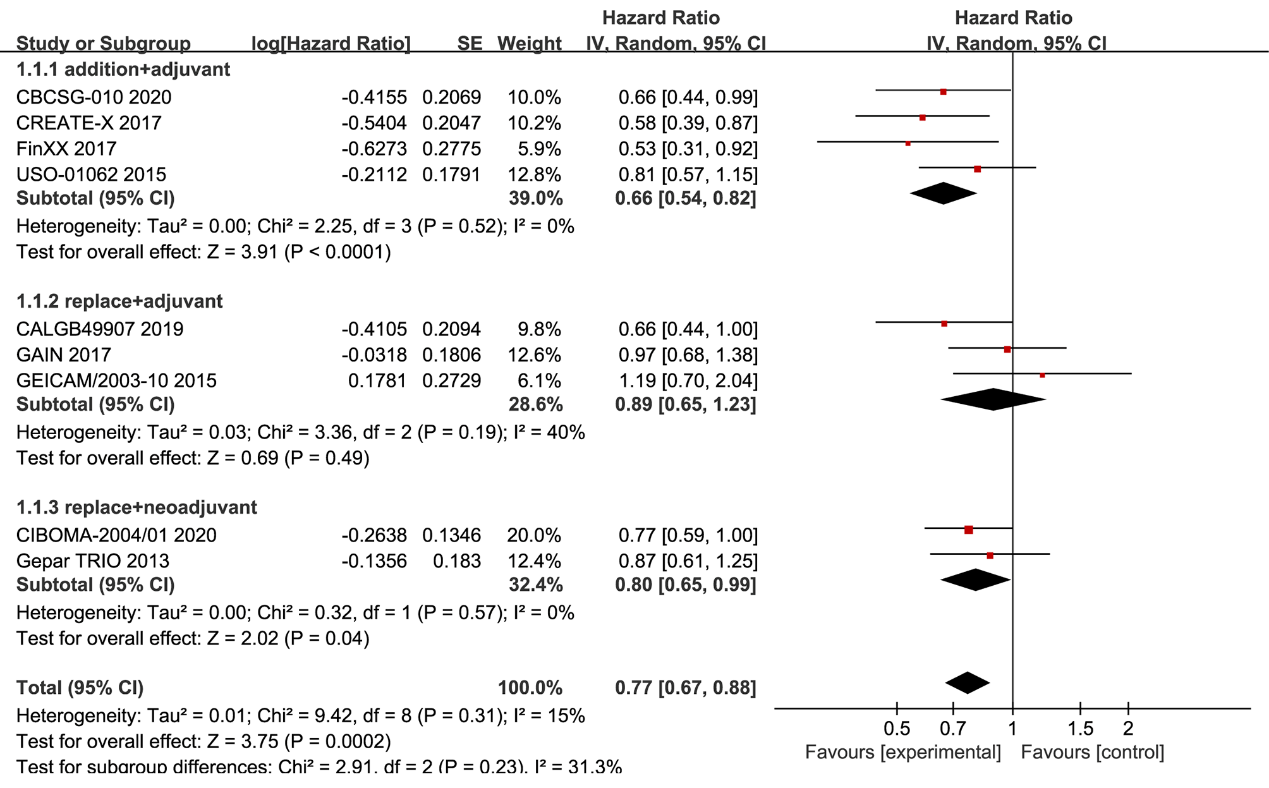


**Figure S2.** Direct meta-analysis of the hazard ratios for disease-free survival based on different pairwise comparisons.


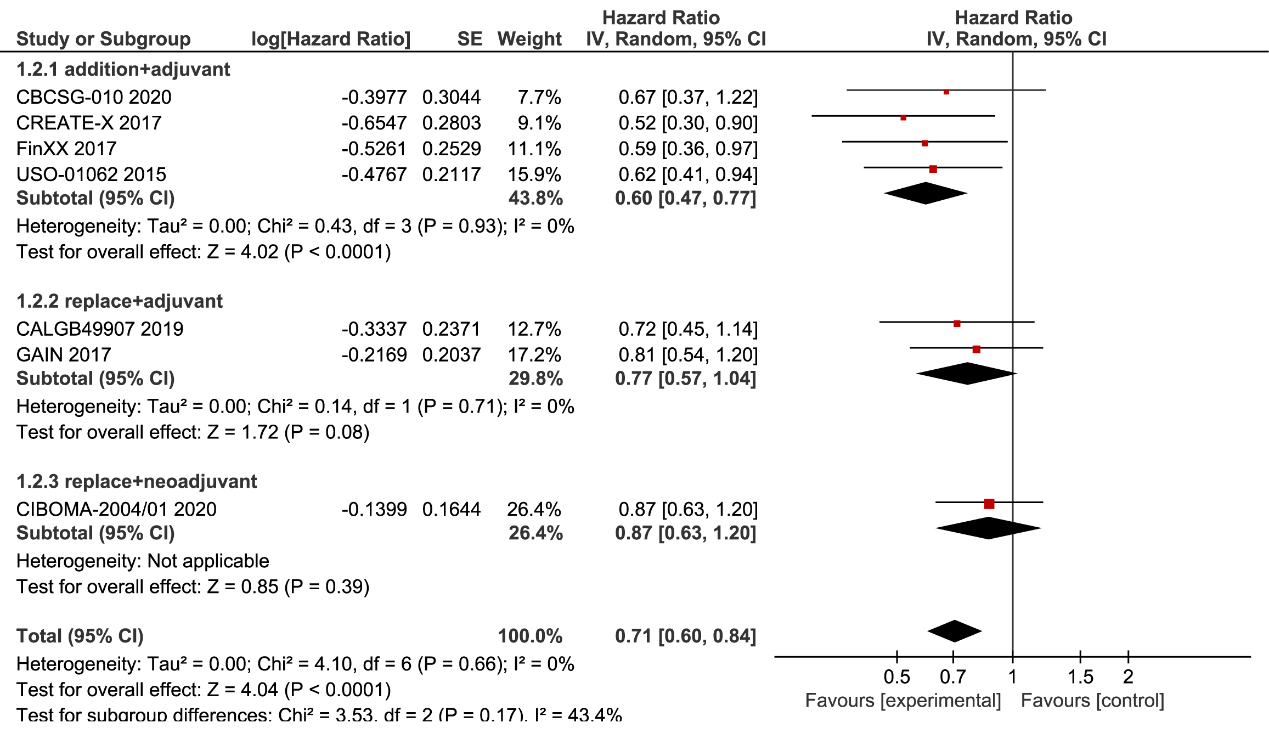


**Figure S3.** Direct meta-analysis of the hazard ratios for overall survival based on different pairwise comparisons.


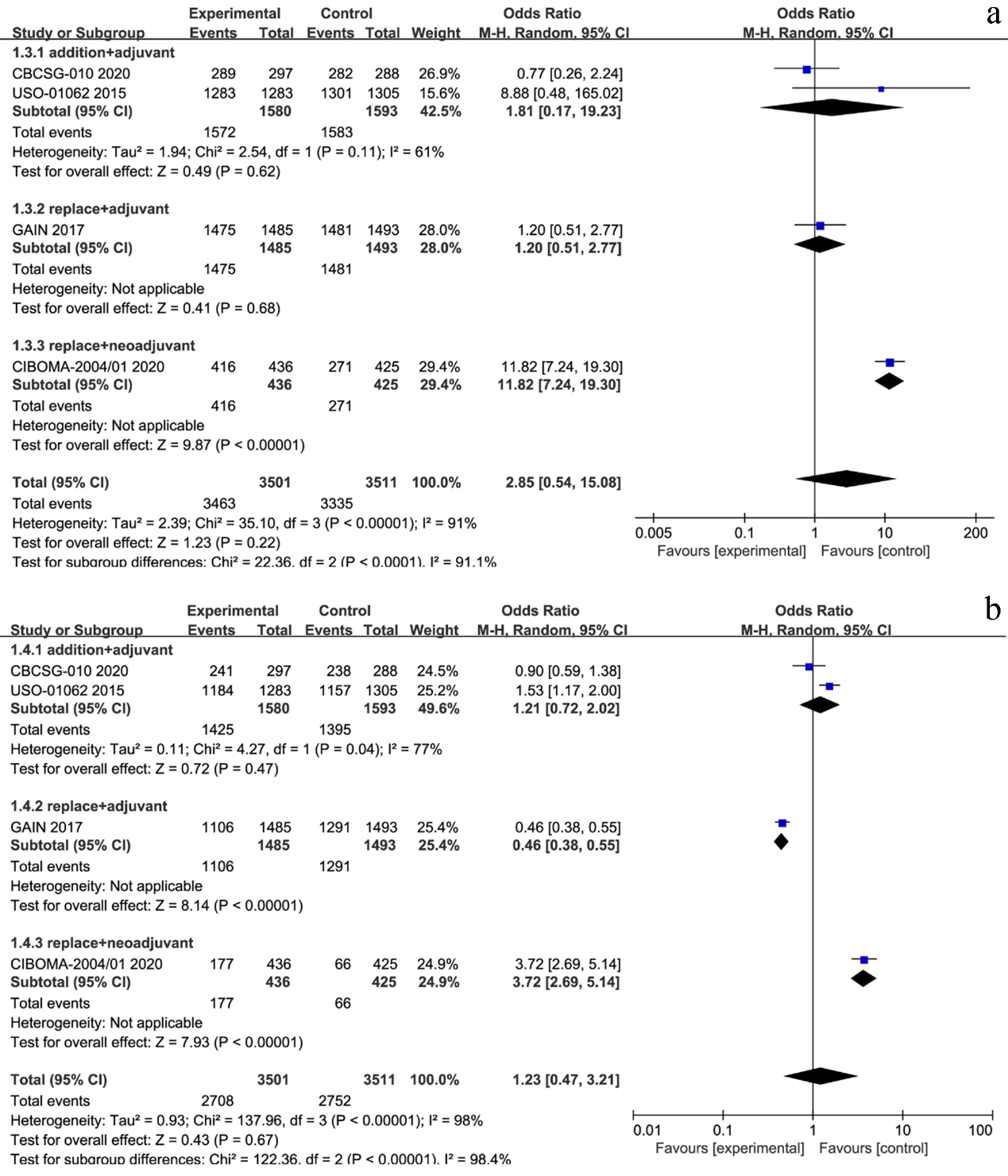


**Figure S4.** Direct meta-analysis of the odds ratios for (A) any adverse events and (B) grade 3-4 adverse events based on different pairwise comparisons.


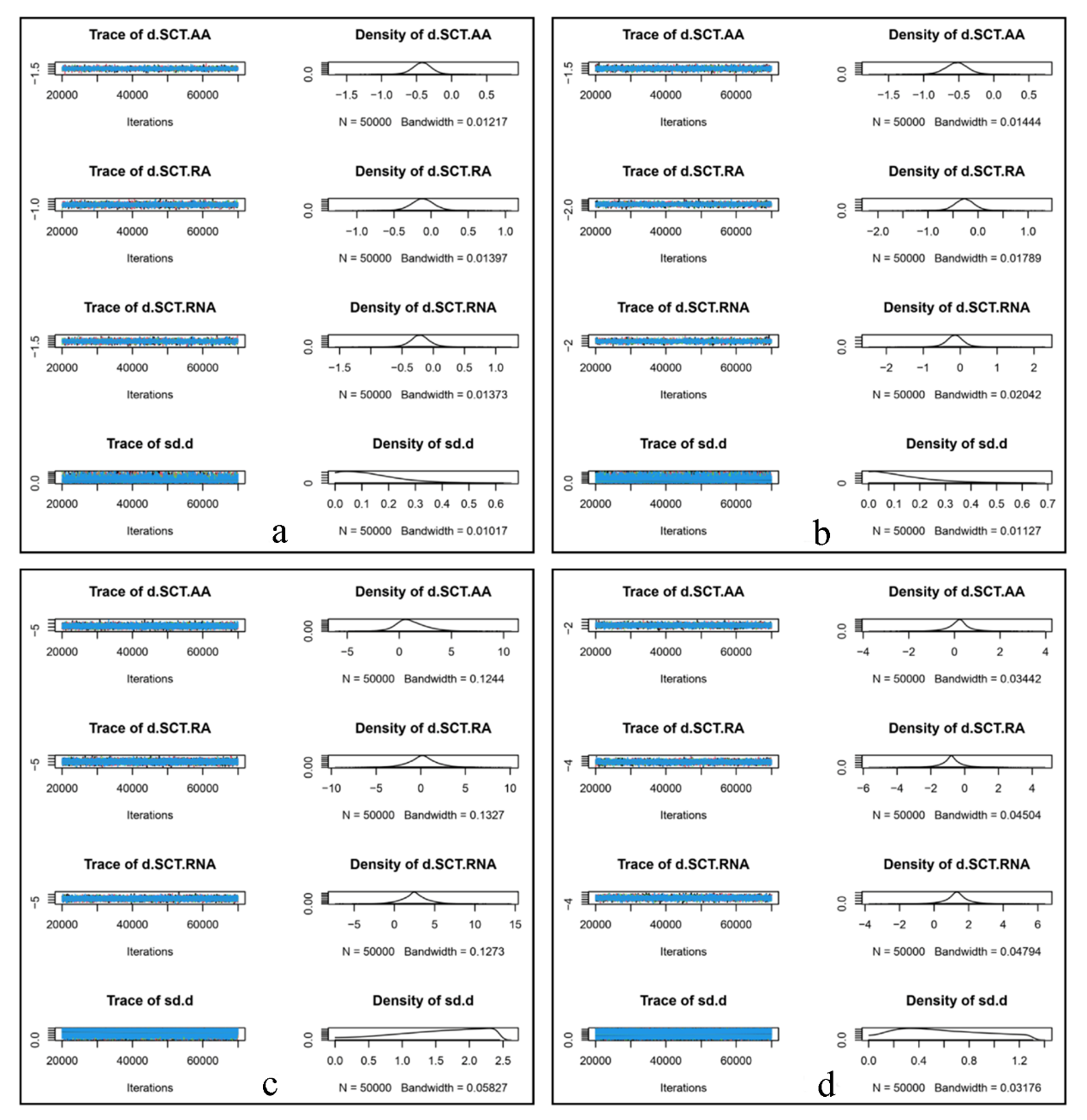


**Figure S5.** Trace and density plots of MCMC simulation for each regime. (a) disease-free survival, (b) overall survival, (c) any adverse events, and (d) grade 3-4 adverse events. AA, addition of capecitabine to adjuvant chemotherapy; RA, replacement of capecitabine to adjuvant chemotherapy; RNA, replacement of capecitabine to neoadjuvant chemotherapy; SCT, standard chemotherapy. MCMC, Markov chain Monte Carlo.
